# Comparison of Reporting Race and Ethnicity in Medical Journals Before and After Implementation of Reporting Guidance, 2019-2022

**DOI:** 10.1001/jamanetworkopen.2023.1706

**Published:** 2023-03-06

**Authors:** Annette Flanagin, Miriam Y. Cintron, Stacy L. Christiansen, Tracy Frey, Timothy Gray, Iris Y. Lo, Roger J. Lewis

**Affiliations:** 1 JAMA Network, Chicago, Illinois; 2 Berry Consultants, Austin, Texas; 3 Harbor-UCLA Medical Center, Torrance, California; 4 David Geffen School of Medicine at UCLA, Los Angeles, California

## Abstract

This cross-sectional study compares race and ethnicity reporting in 3 medical journals before and after implementation of updated guidance on the reporting of race and ethnicity in August 2021.

## Introduction

Previous research has found limited progress in reporting race and socioeconomic status in studies published in medical journals from 2015 to 2019.^1^ This study compared race and ethnicity reporting in 3 medical journals before and after implementation of Updated Guidance on the Reporting of Race and Ethnicity in Medical and Science Journals in August 2021.^2^

## Methods

This cross-sectional study did not include human participants and was determined exempt from ethical review and the requirement for informed consent per US regulation (45 CFR 46) and is reported following the Strengthening the Reporting of Observational Studies in Epidemiology (STROBE) reporting guideline. In this study, all major research articles published in *JAMA*, *JAMA Internal Medicine*, and *JAMA Pediatrics* in January to March 2019, May to July 2021 (immediately before guidance implementation), and January to March 2022 were included. The first 2 periods were identified to serve as approximate historical controls. Articles were assessed independently by 2 reviewers for the following: study included human participants; race and ethnicity, age, sex and gender, and measures of socioeconomic status were reported; where race and ethnicity were reported (eg, abstract, methods, results, tables); number and order of racial and ethnic categories reported; whether the category “other” was included and whether that was defined; and whether the article indicated how race and ethnicity were determined. Comparisons between years were calculated with χ^2^ test for 2-sided *P* values, odds ratios (ORs), and Wald test 95% CIs. Significance was set at *P* = .05. Analyses were conducted using R statistical software version 4.2.0 (R Project for Statistical Computing).

## Results

Of 258 research articles published during the study periods, 249 (96.5%) included human participants and were included in this analysis. In 2019, 49 of 86 articles (57.0%) reported race and ethnicity of study participants, compared with 42 of 77 articles (54.5%) in 2021 and 58 of 86 articles (67.4%) in 2022 (Table). Compared with the proportion of articles reporting race and ethnicity, a higher proportion of articles reported participants’ age and sex or gender, and a lower proportion reported socioeconomic status measures in all years (Table). There were no significant differences over time in article location of reporting race and ethnicity or the proportion of articles that reported racial and ethnic categories (median [IQR], 5 [3-6] racial and ethnic categories reported per articles). Of articles that included “other” as a collective racial and ethnic category, the proportion that defined categories included in “other” was 26.7% in 2019, 70.4% in 2021, and 84.8% in 2022, with a significant difference observed before final guidance implementation in 2021 vs 2019 (OR, 6.53; 95% CI, 2.05-20.76; *P* < .001). A significant difference after reporting guidance implementation was observed for articles listing racial and ethnic categories in alphabetical order (92.6% in 2022 vs 16.7% in 2021; OR, 73.75; 95% CI, 20.15-269.99; *P* < .001) and for articles indicating how race and ethnicity were determined (74.1% in 2022 vs 50.0% in 2021; OR, 2.87; 95% CI, 1.23-6.66; *P* = .01) (Table).

**Table.** Demographic Characteristics Reported in 3 Medical Journals in 2019, 2021, and 2022

Reporting characteristics	No. (%)		2021 vs 2019		2022, No. (%)	2022 vs 2021	
	2019	2021	OR (95% CI)	*P* value		OR (95% CI)	*P* value
Articles with human participants	86 (95.6)	77 (96.3)	NA	NA	86 (97.7)	NA	NA
Demographic characteristics reported							
Age	84 (97.7)	75 (97.4)	0.89 (0.12-6.50)	>.99	86 (100.0)	5.73 (0.27-121.20)^a^	.43
Sex and gender	79 (91.9)	73 (94.8)	1.62 (0.45-5.75)	.45	75 (87.2)	0.37 (0.11-1.23)	.09
Race and ethnicity	49 (57.0)	42 (54.5)	0.91 (0.49-1.68)	.76	58 (67.4)	1.73 (0.91-3.26)	.09
Socioeconomic status measures	32 (37.2)	34 (44.2)	1.33 (0.71-2.50)	.37	37 (43.0)	0.95 (0.51-1.78)	.88
Location of reporting race and ethnicity							
Abstract	16 (18.6)	18 (23.4)	1.33 (0.63-2.85)	.45	19 (22.1)	0.93 (0.45-1.94)	.85
Methods	37 (43.0)	41 (53.2)	1.51 (0.81-2.80)	.19	51 (59.3)	1.28 (0.69-2.38)	.44
Results	33 (38.4)	26 (33.8)	0.82 (0.43-1.56)	.54	40 (46.5)	1.71 (0.90-3.22)	.10
Table	45 (52.3)	40 (51.9)	0.98 (0.53-1.82)	.96	51 (59.3)	1.35 (0.72-2.51)	.35
Race and ethnicity categories							
Reported, median (IQR), No.	4 (3-5)	5 (3-6)	NA	NA	5 (4-6)	NA	NA
Articles reporting categories	48 (55.8)	42 (54.5)	0.95 (0.51-1.76)	.87	54 (62.8)	1.41 (0.75-2.63)	.28
Included “other” category	30 (62.5)	27 (64.3)	1.08 (0.46-2.55)	.86	33 (61.1)	0.87 (0.38-2.01)	.75
Defined categories included in “other”	8 (26.7)	19 (70.4)	6.53 (2.05-20.76)	<.001	28 (84.8)	2.36 (0.67-8.31)	.18
Presented categories in alphabetical order	4 (8.3)	7 (16.7)	2.20 (0.60-8.12)	.23	50 (92.6)	73.75 (20.15-269.99)	<.001
Indicated how race and ethnicity determined	19 (38.8)	21 (50.0)	1.58 (0.69-3.64)	.28	43 (74.1)	2.87 (1.23-6.66)	.01

Abbreviations: OR, odds ratio; NA, not applicable.

^a^ Calculated by adding 0.5 to each cell because of zero count.

## Discussion

In this analysis, higher proportions of articles reported how race and ethnicity were determined and listed categories in alphabetical order in 2022 following the implementation of reporting guidance. This study was limited by the inclusion of only 3 journals from 1 publisher and did not assess differences in reporting by geographic location of the corresponding author of the study or the type of study. The study is also limited by our attempt to include historical controls with the 2 sets of articles that were published before the final guidance was released and the role of unmeasured confounders. The analysis did not determine if changes in reporting occurred during peer review or after acceptance during the editing process, or address potential confounding introduced by the increased awareness of and attention to issues of racism and social justice in academic medicine and society during this study.

In this study, some improvements were observed in the reporting of race and ethnicity in studies published in 3 medical journals between 2019 and 2022 and following implementation of final reporting guidance in 2021. However, race and ethnicity and socioeconomic status were still underreported compared with age, sex, and gender. Further improvement in the reporting of research participant demographics is needed.

## Notes

Supplement.
Data Sharing Statement
