# Supplementary material for: Comparison of Reporting Race and Ethnicity in Medical Journals Before and After Implementation of Reporting Guidance, 2019-2022
Source: JAMA Netw Open. 2023 Mar 6;6(3):e231706. doi: 10.1001/jamanetworkopen.2023.1706 (PMC9989893; doi:10.1001/jamanetworkopen.2023.1706)
Supplement: Supplement. — Data Sharing Statement [file jamanetwopen-e231706-s001.pdf]

Data Sharing Statement

Flanagin. Comparison of Reporting Race and Ethnicity in Medical Journals Before and After Implementation of Reporting Guidance, 2019-2022. *JAMA Netw Open*. Published March 06, 2023. doi:10.1001/jamanetworkopen.2023.1706

Data
Data available: Yes
Data types: Data (not involving human participants)
How to access data: Available from the authors on request after approval for access.
When available: With publication

Supporting Documents
Document types: None

Additional Information
Who can access the data: Researchers whose proposed use of the data has been approved
Types of analyses: Research
Mechanisms of data availability: After approval of a proposal
